# First detection and molecular identification of *Rickettsia massiliae*, a human pathogen, in *Rhipicephalus sanguineus* ticks collected from Southern Taiwan

**DOI:** 10.1371/journal.pntd.0010917

**Published:** 2022-11-11

**Authors:** Li-Lian Chao, Melissa Robinson, You-Fu Liang, Chien-Ming Shih

**Affiliations:** 1 M.Sc. Program in Tropical Medicine, College of Medicine, Kaohsiung Medical University, Kaohsiung, Taiwan; 2 Graduate Institute of Medicine, College of Medicine, Kaohsiung Medical University, Kaohsiung, Taiwan; 3 Department of Medical Research, Kaohsiung Medical University Hospital, Kaohsiung, Taiwan; Creighton University, UNITED STATES

## Abstract

The *Rickettsia massiliae* was firstly detected and identified in *Rhipicephalus sanguineus* ticks infested on dogs in Taiwan. A total of 1154 *Rh*. *sanguineus* ticks collected from 158 dogs of four districts of Tainan city were examined for *Rickettsia* infection by nested-PCR assay targeting the citrate synthase (*gltA*) and outer membrane protein B (*ompB*) genes of *Rickettsia*. The *Rickettsia* infection was detected with a general infection rate of 2.77%, and was detected in male, female and nymphal stage with an infection rate of 2.77%, 3.22% and 1.32%, respectively. Phylogenetic relationships were analyzed by comparing the *gltA* and *ompB* sequences obtained from 9 Taiwan strains and 16 other strains representing 13 genospecies of *Rickettsia*. Results revealed that all Taiwan strains were genetically affiliated to the same clades of *R*. *massiliae* (spotted fever group) and *R*. *felis* (transitional group), and can be discriminated from other genospecies of *Rickettsia*. This study provides the first evidence of *R*. *massiliae*, a pathogenic spotted fever *Rickettsia*, identified in *Rh*. *sanguineus* ticks and highlight the potential threat for the regional transmission of *Rickettsia* infection among humans in Taiwan.

## Author summary

The emerging tick-borne *Rickettsia* pathogens have a major impact on human health worldwide. In previous studies, *R*. *massiliae* has been isolated and recognized as a human pathogen in Italy, southern France and Argentina. The first isolation of *R*. *massiliae* from *Rhipicephalus* ticks was reported in Maresille and was further found in Greece, Spain, Portugul, Switzerland, Sicily, Central Africa, and Mali. Our previous studies have found various *Rickettsia* (*R*. *parkeri*-like, *R*. *honei*, and *R*. *felis*) in *Ixodes granulatus* and *Rh*. *sanguineus* ticks in Taiwan area. In this study, *R*. *massiliae* was firstly detected in *Rh*. *sanguineus* ticks infested on dogs. As a companion animal for humans, the first detection of human pathogenic strain of *R*. *massiliae* in *Rh*. *sanguineus* ticks from dogs may imply the potential threat for the regional transmission of *Rickettsia* infection among humans in Taiwan.

## Introduction

The *Rhipicephalus sanguineus* tick is a haematophagus arthropod and is observed as the most common ectoparasite of dogs around the world [1, 2]. Previous investigations demonstrated that the existence of at least two distinguished groups (tropical vs temperate lineage) of *Rh*. *sanguineus* sensu lato ticks based on the genetic comparison of 16S mitochondrial rRNA gene [3, 4]. In addition, *Rh*. *sanguineus* has been attributed to the main vector for the transmission of *Babesia*, *Ehrlichia*, and *Rickettsia* among humans and animals [5–7]. Due to the increasing detection of *B*. *vogeli*, *B*. *gibsoni*, *Anaplsma platys*, *R*. *felis* and canine babesiosis in Taiwan [8–12], the medical and veterinary importance of *Rh*. *sanguineus* ticks raise the research attention on this tick species. Although the *Rh*. *sanguineus* ticks had been identified as the potential vector ticks for a variety of pathogens in Taiwan, there has no research confirming the genetic identification of *Rickettsia massiliae*, a pathogenic strain for human infection, in this tick species in Taiwan.

The genus *Rickettsia* composed of approximately 27 species of obligate intracellular gram-negative bacteria that can be classified into four major groups and the spotted fever group (SFG) is responsible for the most *Rickettsia* infections in humans [13–15]. Various arthropods including tick, flea, mite and louse, may serve as vector for *Rickettsia* transmission. However, the Ixodid ticks may serve as the primary vectors and reservoirs of amplifying hosts for *Rickettsia* agents [16]. Except the flea-borne *R*. *felis* and mite-borne *R*. *akari*, the SFG rickettsiae are mainly transmitted by vector ticks and some of these ticks can transmit the *Rickettsia* agents through the transovarial and transstadial pathways [17]. During the past decades, *Rickettsia* infections become a global threat of emerging tick-borne diseases [18, 19], and the *R*. *massiliae* and *R*. *felis* have been identified as the pathogenic strains for infecting humans [20–26]. In addition, many validated SFG rickettsial species have been discovered in Australia, Central and South America, and Asia [27–37]. In Taiwan, human serosurvey of *Rickettsia* infections had been attributed to the agent of *R*. *felis* in southern Taiwan [38]. However, there is no confirming evidence of human isolate and vector tick that is responsible for the transmission of *Rickettsia* infections in Taiwan. Thus, epidemiological survey on tick-borne rickettsiae in *Rh*. *sanguineus* ticks is crucial to understand the potential threat of emerging tick-borne *Rickettsia* infections in Taiwan.

Molecular approach based on the genetic variance at the individual base-pair level gives much more direct pathway for measuring the genetic diversity between and within species of *Rickettsia* [39, 40]. Previous studies based on the molecular marker of citrate synthase (*gltA*) and outer membrane protein B (*ompB*) genes have concluded that it is sufficiently informative for the analysis of evolutionary relationships between the genetic diversity of *Rickettsia* species among various vectors and hosts [31–40]. Thus, molecular detection and genetic analysis based on the phylogenetic analysis of *gltA* and *ompB* genes have made possible in facilitating the identification and discrimination of *Rickettsia* species within ticks.

It may be that the *Rickettsia* infection in *Rh*. *sanguineus* ticks of Taiwan is genetically distinct genospecies, as compared with the existing common genospeciess of *Rickettsia* around the world. Thus, the objectives of this study intend to investigate the prevalence of *Rickettsia* infection in *Rh*. *sanguineus* ticks collected from Taiwan and to determine the phylogenetic relationships between and within the genospecies of *Rickettsia* in these ticks. The genetic affiliation of *Rickettsia* strains detected in *Rh*. *sanguineus* ticks of Taiwan was analyzed by comparing their differential nucleotide composition with other *Rickettsia* strains identified from various biological and geographical sources which have been documented in GenBank.

## Methods

### Tick collection and species identification

**Fig 1:**
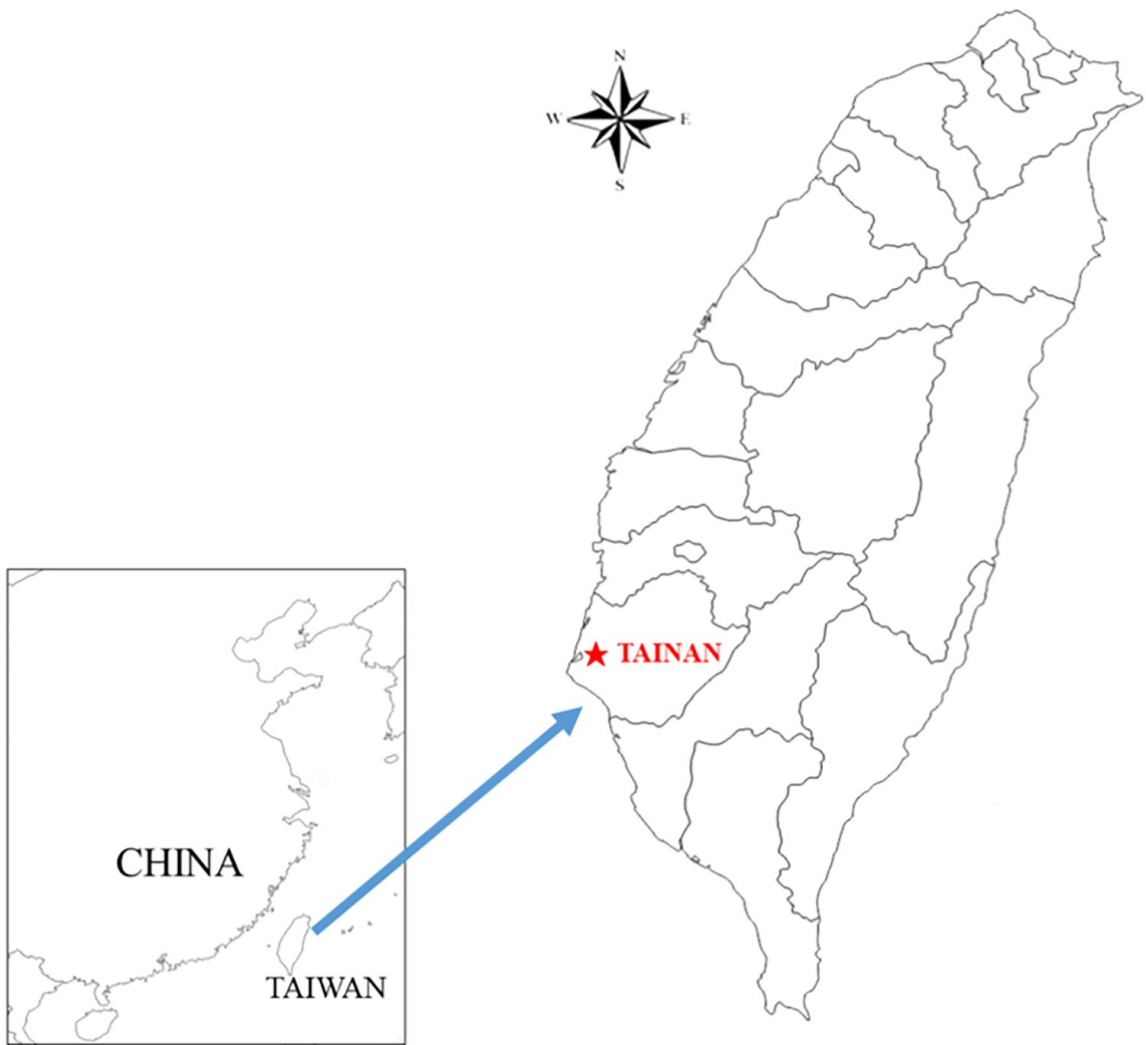
Map of Taiwan showing the collection site (★) for ticks from Tainan City. This map is adapted from free maps (East China map: https://d-maps.com/carte.php?num_car=175&lang=en and Taiwan map; https://d-maps.com/carte.php?num_car=651&lang=en]).

All specimens of *Rh*. *sanguineus* ticks used in this study were collected from 158 dogs of four districts of Tainan city that include Yong-Kang (YK), Ren-De (RD), East District (ED) and South District (SD) in southern Taiwan (**Fig 1**). All these dogs were handled by veterinary practitioner for collecting the attached ticks. All these ticks were subsequently cleaned and stored in separate glass vials containing 75% ethanol. All tick specimens of *Rh*. *sanguineus* were identified to the species level on the basis of their morphological characteristics [2, 41] and genetic identification was also performed based on the mitochondrial 16S rRNA gene, as described previously [9]. Briefly, the external features of the *Rh*. *sanguineus* ticks were recorded by using a stereo-microscope (SMZ 1500, Nikon, Tokyo, Japan) equipped with a fiber lamp and photographed for species identification.

### DNA extraction from tick specimens

Total genomic DNA was extracted from individual tick specimens used in this study. Briefly, tick specimens were cleaned by sonication for 3–5 min in 75% ethanol solution and then washed twice in sterile distilled water. Afterwards, the individual tick specimen was homogenized in a microcentrifuge tube filled with 180-μL lysing buffer solution (DNeasy Blood &Tissue Kit, catalogue no. 69506, Qiagen, Taipei, Taiwan) and then homogenized with a TissueLyser II apparatus (catalogue no. 85300, Qiagen, Germany), instructed by the manufacturer. The homogenate was centrifuged at room temperature and the supernatant fluid was further processed by a DNeasy Blood & Tissue Kit (catalogue no. 69506, Qiagen, Taipei, Taiwan), as instructed by the manufacturer. After filtration with the kit, the filtrated fluid was collected and the DNA concentration was determined spectrophotometrically with a DNA calculator (Epoch, Biotek, USA) and the extracted DNA is stored at -80°C for further investigations [9].

### DNA amplification by nested polymerase chain reaction

DNA samples extracted from each tick specimens were used as a template for PCR amplification. Two primer sets based on the citrate synthase gene (*gltA*) were used for amplification. Initially, the primer set of RpCS.877p (5’-GGGGGCCTGCTCACGGCGG-3’) and RpCs.1258n (5’-ATTGCAAAAAGTACAGTGAACA-3’) was used to amplify the primary product of *gltA*. Nested PCR was then performed using the species-specific primer sets: RpCS.896p (5’-GGCTAATGAAGCAGTGATAA-3’) and RpCS.1233n (5’-GCGACGGTATACCCATAGC-3’) for amplifying a product approximately 338-bp [42]. All PCR reagents and Taq polymerase were obtained and used as recommended by the supplier (Takara Shuzo Co., Ltd., Japan). Briefly, each 25-μl reaction mixture containing 3-μl DNA template, 1.5-μl forward and reverse primers, 2.5-μl 10X PCR buffer (Mg^2+^), 2-μl dNTP mixture (10 mM each), 1 unit of Taq DNA polymerase and filled-up with adequate volume of ddH_2_O. In contrast, adequate amounts of sterile distilled water were added for serving as a negative control. PCR amplification was performed with a thermocycler (Veriti, Applied Bioosystems, Taipei, Taiwan) and was denaturation at 95°C for 5 min and then amplified for 35 cycles with the conditions of denaturation at 95°C for 30 sec, annealing at 54°C for 30 sec, extension at 72°C for 1 min, and followed by a final extension step at 72°C for 3 min. For the nested-PCR, the following conditions were used: denaturation at 95°C for 5 min and then amplified for 40 cycles with the conditions of denaturation at 95°C for 30 sec, annealing at 50°C for 30 sec, extension at 72°C for 1 min, and followed by a final extension step at 72°C for 3 min.

For outer membrane protein B gene (*ompB*), the primer sets of rompB-OF (5’-GTAACCGGAAGTAATCGTTCGTAA-3’) and rompB-OR (5’-GCTTTATAACCAGCTAAACCACC-3’) was used to amplify the primary product of *ompB*. Nested PCR was then performed using the species-specific primer sets: rompB SPG-IF (5’-GTTTAATACGTGCTGCTAACCAA-3’) and rompB SPG/TG-IR (5’-GGTTTGGCCCATATACCATAAG-3’) for amplifying a product approximately 420-bp [42]. The PCR conditions were used same as *gltA* amplification except the annealing temperature of 50°C and 52°C for the initial and nested PCR cycles, respectively.

All amplified PCR products were electrophoresed on 1.5% agarose gels in Tris-Borate-EDTA (TBE) buffer and visualized under ultraviolet (UV) light after staining with ethidium bromide. A 100-bp DNA ladder (GeneRuler, Thermo Scientific, Taiwan) was used as the standard marker for comparison. A negative control of distilled water was included in parallel with each amplification.

### Sequence alignments and phylogenetic analysis

Approximately 10-μl of each selected samples with clear bands on the agarose gel was submitted for DNA sequencing (Mission Biotech Co., Ltd., Taiwan). After purification (QIAquick PCR Purification Kit, catalog No. 28104), sequencing reaction was performed with 25 cycles under the same conditions and same primer set of nested amplification by dye-deoxy terminator reaction method using the Big Dye Terminator Cycle Sequencing Kit in an ABI Prism 377–96 DNA Sequencer (Applied Biosystems, Foster City, CA, USA). The resulting sequences were initially edited by BioEdit software (V5.3) and aligned with the CLUSTAL W software [43]. Thereafter, the aligned sequences of *Rickettsia gltA* gene from 9 Taiwan strains were analyzed by comparing with other 16 strains of *Rickettsia* sequences from the different biologiical and geographical origin that are available from GenBank. Further analysis based on *ompB* gene of 8 Taiwan strains belonging to the SFG *Rickettsia* was performed by comparing with other 15 strains of *Rickettsia* sequences documented in GenBank. Phylogenetic analysis was performed by neighbour-joining (NJ) compared with maximum likelihood (ML) methods to estimate the phylogeny of the entire alignment using MEGA X software package [44]. The genetic distance values of inter- and intra-species variations were also analyzed by the Kimura two-parameter model [45]. All phylogenetic trees were constructed and performed with 1000 bootstrap replications to evaluate the reliability of the construction, as described previously [46].

### Ethical approval

The collection of ticks from dogs was assistant by veterinary practitioners and approved by the Institutional Animal Care and Use Committee (IACUC) of Kaohsiung Medical University (IACUC Approval No: 106142).

## Results

### Detection of *Rickettsia* infection in *Rh*. *sanguineus* ticks

**Fig 2:**
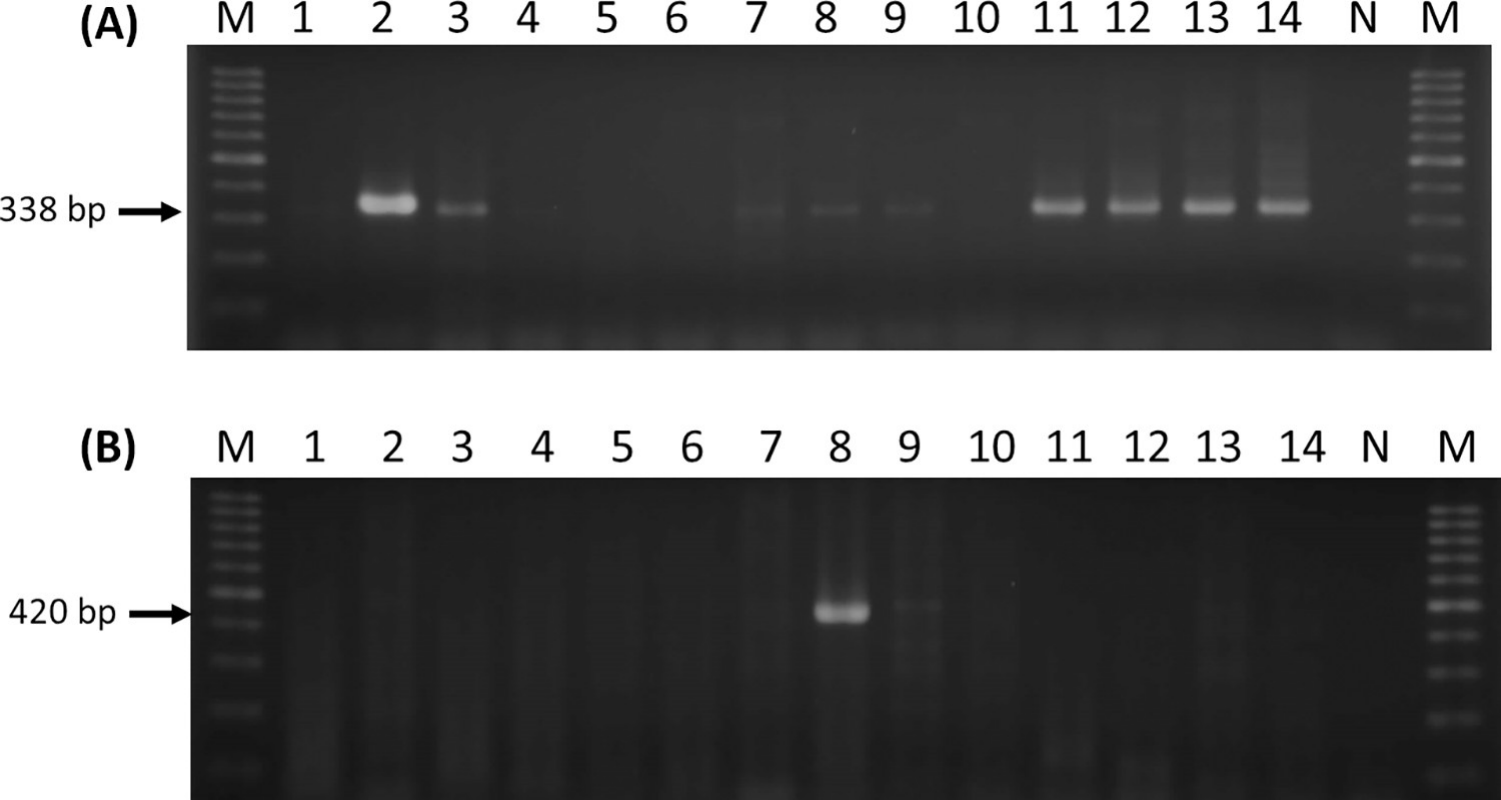
Molecular detection of *Rickettsia* infection in *Rhipicephalus sanguineus* ticks from Tainan City of Taiwan by nested PCR assay targeting (A) citrate synthase (*gltA*) and (B) outer membrane protein B (*ompB*) genes. M, 100 bp DNA marker; 1–14, sample number; N, negative control. The expected PCR product of 338 bp and 420 bp for *gltA* and *ompB*, respectively.

The existence of *Rickettsia* in *Rh*. *sanguineus* ticks was detected by nested PCR assay targeting the *gltA* and *ompB* genes (**Fig 2**). In general, a total of 2.77% (32/1154) *Rh*. *sanguineus* ticks were detected with *Rickettsia* infection by targeting the *gltA* gene. Further analysis of 32 *gltA* positive specimens by targeting the *ompB* gene revealed 19 positive *Rickettsia* detection and only 8 specimens were positive detected by both *gltA* and *ompB* genes. According to the life stage of ticks, the *Rickettsia* infection was detected in males, females and nymphs of *Rh*. *sanguineus* ticks with an infection rate of 2.77%, 3.22% and 1.32%, respectively (**Table 1**). The highest geographical prevalence of *Rickettsia* infection was detected in Yong-Kang (4.26%) and East District (3.87%).

**Table 1:** Molecular detection of *Rickettsia* infection^a^ in *Rhipicephalus sanguineus* ticks of Taiwan.

Site of collection^b^	Rickettsia infection detected in various life-stage of tick			
	Male	Female	Nymph	Total
	No. infected /no. examined (%)	No. infected /no. examined (%)	No. infected /no. examined (%)	No. infected /no. examined (%)
YK	5/153 (3.27)	7/115 (6.09)	0/14 (0)	12/282 (4.26)
RD	4/234 (1.71)	6/256 (2.34)	2/119 (1.68)	12/609 (1.97)
ED	4/78 (5.13)	3/84 (3.57)	0/19 (0)	7/181 (3.87)
SD	1/40 (2.50)	0/42 (0)	0/0 (0)	1/82 (1.22)
**Total**	**14/505 (2.77)**	**16/497 (3.22)**	**2/152 (1.32)**	**32/1154 (2.77)**

^a^Nested-PCR assay targeting the citrate synthase (*gltA*) and outer membrane protein B (*ompB*) genes.

^b^Districts of YK (Yong-Kang), RD (Ren-De), ED (East District) and SD (South District) of Tainan City, Taiwan.

### Sequence alignment and genetic analysis of *Rickettsia* in *Rh*. *sanguineus* ticks

To clarify the genetic identity of *Rickettsia* in *Rh*. *sanguineus* ticks of Taiwan, the sequences of *gltA* and *ompB* gene fragments from 9 Taiwan strains of *Rickettsia* performed by this study were aligned and compared with the downloaded sequences of 16 other *Rickettsia* strains from different biological and geographical origin documented in GenBank. Results indicate that all these *Rickettsia* strains detected in *Rh*. *sanguineus* ticks of Taiwan were genetically affiliated to the genospecies of *R*. *massiliae* and *R*. *felis* with the highly sequence similarity of 99.01–99.68% and 99.01–99.67% respectively (**Table 2**). In addition, intra- and inter-species analysis based on the genetic distance (GD) values of *gltA* gene indicated a lower levels (GD<0.013 and <0.059 for *R*. *massiliae* and *R*. *felis*) of genetic divergence within the *Rickettsia* strains of Taiwan, as compared with the type strain of *R*. *massiliae* and *R*. *felis*, respectively (Table 3). Further analysis based on the GD values of *ompB* gene indicated a lower levels (GD<0.013 and <0.052 for *R*. *massiliae* and *R*. *felis*) of genetic divergence within the *Rickettsia* strains of Taiwan, as compared with the type strain of *R*. *massiliae* and *R*. *felis*, respectively (**Table 3**).

**Table 2:** Intra- and Inter-species analysis of genetic distance values^a^ based on the *gltA* gene sequences among various *Rickettsia* strains.

*Rickettsia* strains	1	2	3	4	5	6	7	8	9	10	11	12	13	14	15	16	17
1. *Rickettsia massiliae* (KJ663741)	-																
2. RS-TN-RD-10702-M14 (**Taiwan**)	0.003	-															
3. RS-TN-YK-10607-M10 (**Taiwan**)	0.003	0.003	-														
4. RS-TN-ED-10703-PEF5 (**Taiwan**)	0.010	0.010	0.007	-													
5. RS-TN-YK-10608-PEF1 (**Taiwan**)	0.013	0.013	0.010	0.010	-												
6. *Rickettsia felis* (MG818715)	0.048	0.048	0.048	0.048	0.056	-											
7. RS-TN-RD-10606-N3 (**Taiwan**)	0.048	0.048	0.048	0.048	0.056	0.059	-										
8. RS-TN-RD-10704-PEF22(**Taiwan**)	0.048	0.048	0.048	0.048	0.056	0.059	0.000	-									
9. RS-TN-RD-10606-M23 (**Taiwan**)	0.048	0.048	0.048	0.048	0.056	0.059	0.000	0.000	-								
10. RS-TN-SD-10608-M40 (**Taiwan**)	0.056	0.056	0.056	0.056	0.048	0.052	0.007	0.007	0.007	-							
11. RS-TN-YK-10612-PEF9(**Taiwan**)	0.056	0.056	0.056	0.056	0.048	0.052	0.007	0.007	0.007	0.007	-						
12. *Rickettsia Japonica* (U59724)	0.013	0.013	0.017	0.017	0.024	0.027	0.041	0.041	0.041	0.041	0.041	-					
13. *Rickettsia honei* (AF022817)	0.020	0.020	0.024	0.024	0.031	0.034	0.048	0.048	0.048	0.048	0.048	0.055	-				
14. *Rickettsia rickettsii* (U59729)	0.027	0.027	0.031	0.031	0.038	0.042	0.052	0.052	0.052	0.052	0.052	0.060	0.060	-			
15. *Rickettsia akari* (U59717)	0.055	0.055	0.055	0.055	0.063	0.067	0.034	0.034	0.034	0.034	0.034	0.041	0.041	0.055	-		
16. *Rickettsia typhi* (U59714)	0.089	0.089	0.089	0.089	0.097	0.101	0.085	0.085	0.085	0.085	0.085	0.092	0.092	0.089	0.089	-	
17. *Rickettsia bellii* (U59716)	0.149	0.149	0.149	0.149	0.158	0.162	0.165	0.165	0.165	0.165	0.165	0.175	0.175	0.157	0.157	0.178	-

^a^The pairwise distance calculation was performed by the method of Kimura 2-parameter, as implemented in MEGA X (Kumar et al., 2018).

**Table 3:** Intra- and Inter-species analysis of genetic distance values^a^ based on the *ompB* gene sequences among various *Rickettsia* strains.

*Rickettsia* strains	1	2	3	4	5	6	7	8	9	10	11	12	13	14	15	16	17
1. *Rickettsia massiliae* (KJ663753)	-																
2. RS-TN-ED-10703-PEF5 (**Taiwan**)	0.005	-															
3. RS-TN-YK-10607-M10 (**Taiwan**)	0.005	0.010	-														
4. RS-TN-YK-10608-PEF1 (**Taiwan**)	0.008	0.013	0.008	-													
5. RS-TN-RD-10702-M14 (**Taiwan**)	0.013	0.018	0.013	0.013	-												
6. RS-TN-YK-10607-PEF3 (**Taiwan**)	0.013	0.018	0.013	0.013	0.005	-											
7. *Rickettsia conorii* (JN182798)	0.021	0.021	0.024	0.024	0.024	0.021	-										
8. *Rickettsia honei* (AF123724)	0.029	0.029	0.032	0.032	0.032	0.032	0.032	-									
9. *Rickettsia rickettsii* (X1635)	0.037	0.037	0.040	0.040	0.040	0.040	0.040	0.016	-								
10. *Rickettsia japonica* (AF123713)	0.032	0.032	0.035	0.035	0.035	0.035	0.035	0.027	0.038	-							
11. *Rickettsia akari* (AF123707)	0.073	0.067	0.076	0.076	0.076	0.076	0.076	0.069	0.079	0.084	-						
12. *Rickettsia felis* (HQ236389)	0.070	0.070	0.073	0.073	0.073	0.073	0.076	0.059	0.073	0.081	0.081	-					
13. RS-TN-RD-10606-M23 (**Taiwan**)	0.071	0.071	0.068	0.068	0.068	0.065	0.068	0.055	0.065	0.072	0.072	0.046	-				
14. RS-TN-RD-10704-PEF22 (**Taiwan**)	0.074	0.074	0.071	0.071	0.071	0.068	0.071	0.055	0.071	0.078	0.078	0.051	0.011	-			
15. RS-TN-YK-10612-PEF9 (**Taiwan**)	0.077	0.077	0.074	0.074	0.074	0.071	0.074	0.061	0.071	0.079	0.079	0.052	0.011	0.005	-		
16. *Rickettsia typhi* (HQ236390)	0.375	0.365	0.379	0.379	0.379	0.379	0.385	0.378	0.362	0.391	0.391	0.374	0.346	0.354	0.357	-	
17 *Rickettsia bellii* (AY970508)	0.522	0.511	0.524	0.524	0.518	0.526	0.524	0.501	0.492	0.501	0.501	0.508	0.496	0.501	0.495	0.518	-

^a^The pairwise distance calculation was performed by the method of Kimura 2-parameter, as implemented in MEGA X (Kumar et al., 2018).

### Nucleotide sequence accession numbers

The nucleotide sequences of PCR-amplified *gltA* gene of 9 *Rickettsia* strains from *Rh*. *sanguineus* ticks of Taiwan determined in this study have been registered and assigned the following GenBank accession numbers: RS-TN-RD-10606-M23 (ON093122), RS-TN-RD-10606-N3 (ON0931233), RS-TN-RD-10704-PEF22 (ON093124), RS-TN-SD-10608-M40 (ON093125), RS-TN-YK-10612-PEF9 (ON093126), RS-TN-ED-10703-PEF5 (ON093127), RS-TN-RD-10702-M14 (ON093128), RS-TN-YK-10607-M10 (ON093129), and RS-TN-YK-10608-PEF1 (ON093130). The GenBank accession numbers for the PCR-amplified *ompB* gene of 8 *Rickettsia* strains from *Rh*. *sanguineus* ticks of Taiwan were also assigned as: RS-TN-RD-10606-M23 (ON093131), RS-TN-RD-10704-PEF22 (ON093132), RS-TN-YK-10612-PEF9 (ON093133), RS-TN-ED-10703-PEF5 (ON093134), RS-TN-RD-10702-M14 (ON093135), RS-TN-YK-10607-M10 (ON093136), RS-TN-YK-10607-PEF3 (ON093137), and RS-TN-YK-10608-PEF1 (ON093138). For phylogenetic analysis, the nucleotide sequences of *gltA* and *ompB* genes from other 19 and 15 *Rickettsia* strains were included for comparison, respectively. Their GenBank accession numbers are shown in **S1 Table**.

### Phylogenetic analysis of *Rickettsia* detected in *Rh*. *sanguineus* ticks

**Fig 3:**
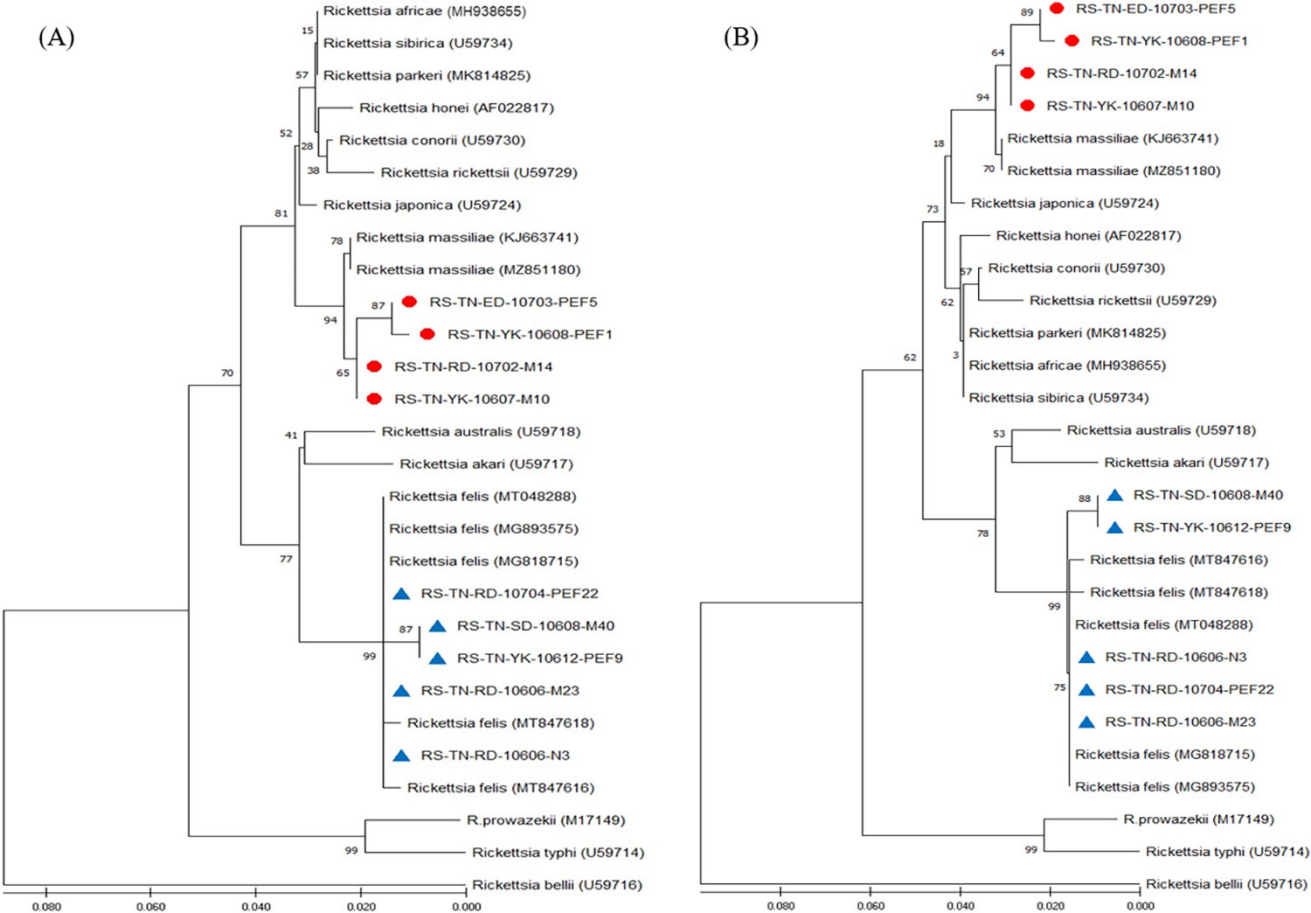
Phylogenetic relationships based on the citrate synthase (*gltA*) gene. Sequences of *Rickettsia* between 9 specimens (indicated as ● & ▲) detected in *Rhipicephalus sanguineus* ticks from Tainan City of Taiwan and 19 other *Rickettsia* specimens identified from various biological and geographical origins. *The Tainan strains were genetically affiliated with the R*. *massiliae* (indicated as ●) and *R*. *felis* (indicated as ▲), respectively. The trees were constructed and analyzed by **(A)** neighbour-joining and **(B)** maximum likelihood methods using 1000 bootstraps replicates. Numbers at the nodes indicate the percentages of reliability of each branch of the tree. Branch length is drawn proportional to the estimated sequence divergence.

**Fig 4:**
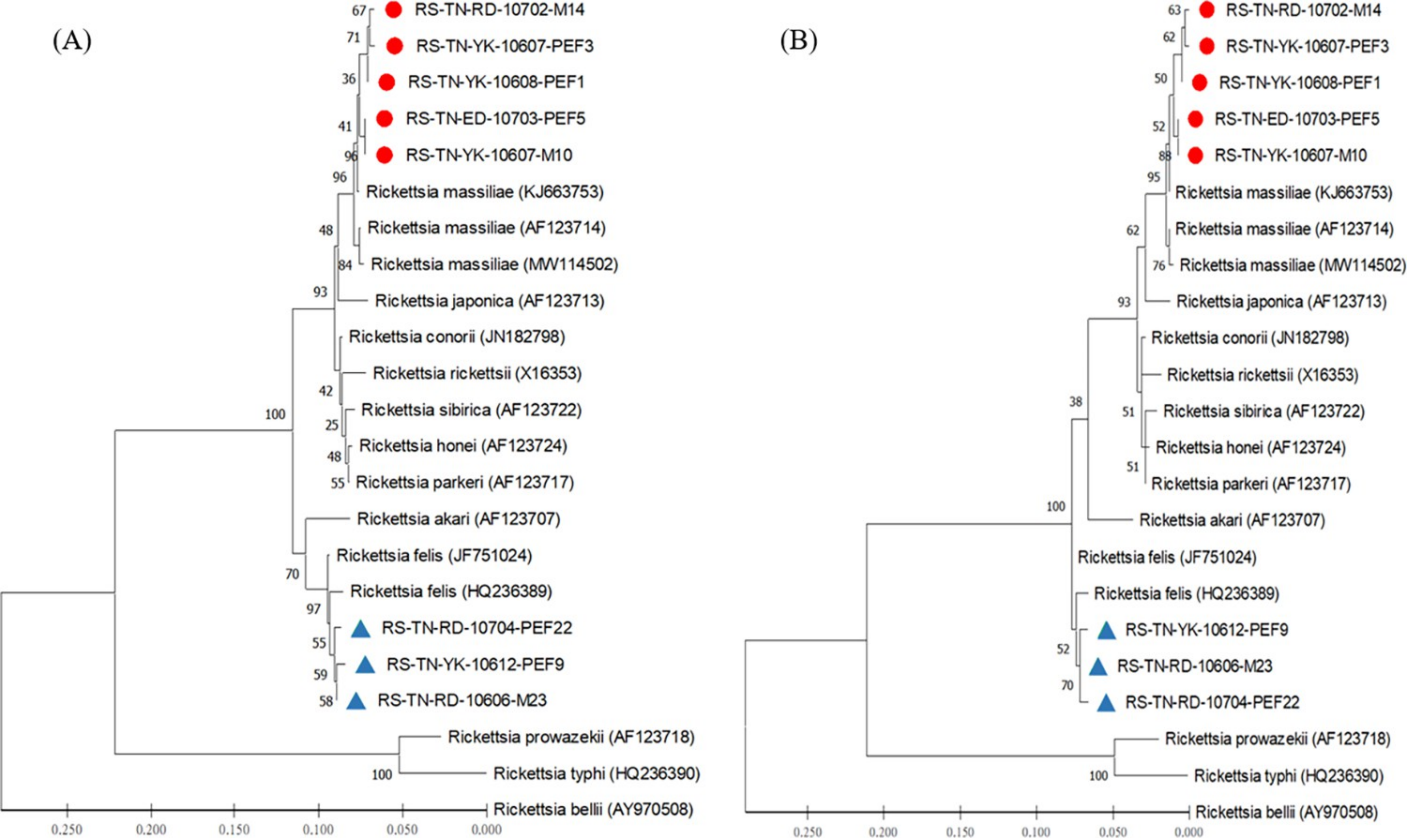
Phylogenetic relationships based on the outer membrane protein B (*ompB*) gene. Sequences of *Rickettsia* between 8 specimens (indicated as ● & ▲) detected in *Rhipicephalus sanguineus* ticks from Tainan City of Taiwan and 15 other *Rickettsia* specimens identified from various biological and geographical origins. The Tainan strains were genetically affiliated with the *R*. *massiliae* (indicated as ●) and *R*. *felis* (indicated as ▲), respectively. The trees were constructed and analyzed by **(A)** neighbour-joining and **(B)** maximum likelihood methods using 1000 bootstraps replicates. Numbers at the nodes indicate the percentages of reliability of each branch of the tree. Branch length is drawn proportional to the estimated sequence divergence.

Phylogenetic relationships based on the sequence alignment of *gltA* and *ompB* genes were performed to demonstrate the genetic relationships among 28 and 23 strains of *Rickettsia* investigated in this study, respectively. Phylogenetic trees constructed by neighbour-joining (NJ) and maximum likelihood (ML) methods were used to analyze the phylogenetic relationships of *Rickettsia* strains. Bootstrap analysis was used to analyze the repeatability of the clustering of specimens represented in phylogenetic trees. Results showed congruent basal topologies with nine major clades of *Rickettsia* that can be easily distinguished by *gltA* analysis (**Fig 3A and 3B**) and were congruent by *ompB* analysis (**Fig 4A and 4B**). In general, all these *Rickettsia* strains from Taiwan constitute a monophyletic clade closely affiliated to the genospecies of *R*. *massiliae* and *R*. *felis*, respectively. These results reveal a lower genetic divergence within the same genospecies of *Rickettsia* detected in *Rh*. *sanguineus* ticks from Taiwan, but a higher genetic variations from other group of *Rickettsia* detected in different biological and geographical origins.

### Genetic identification of *Rh*. *sanguineus* ticks collected from southern Taiwan

**Fig 5:**
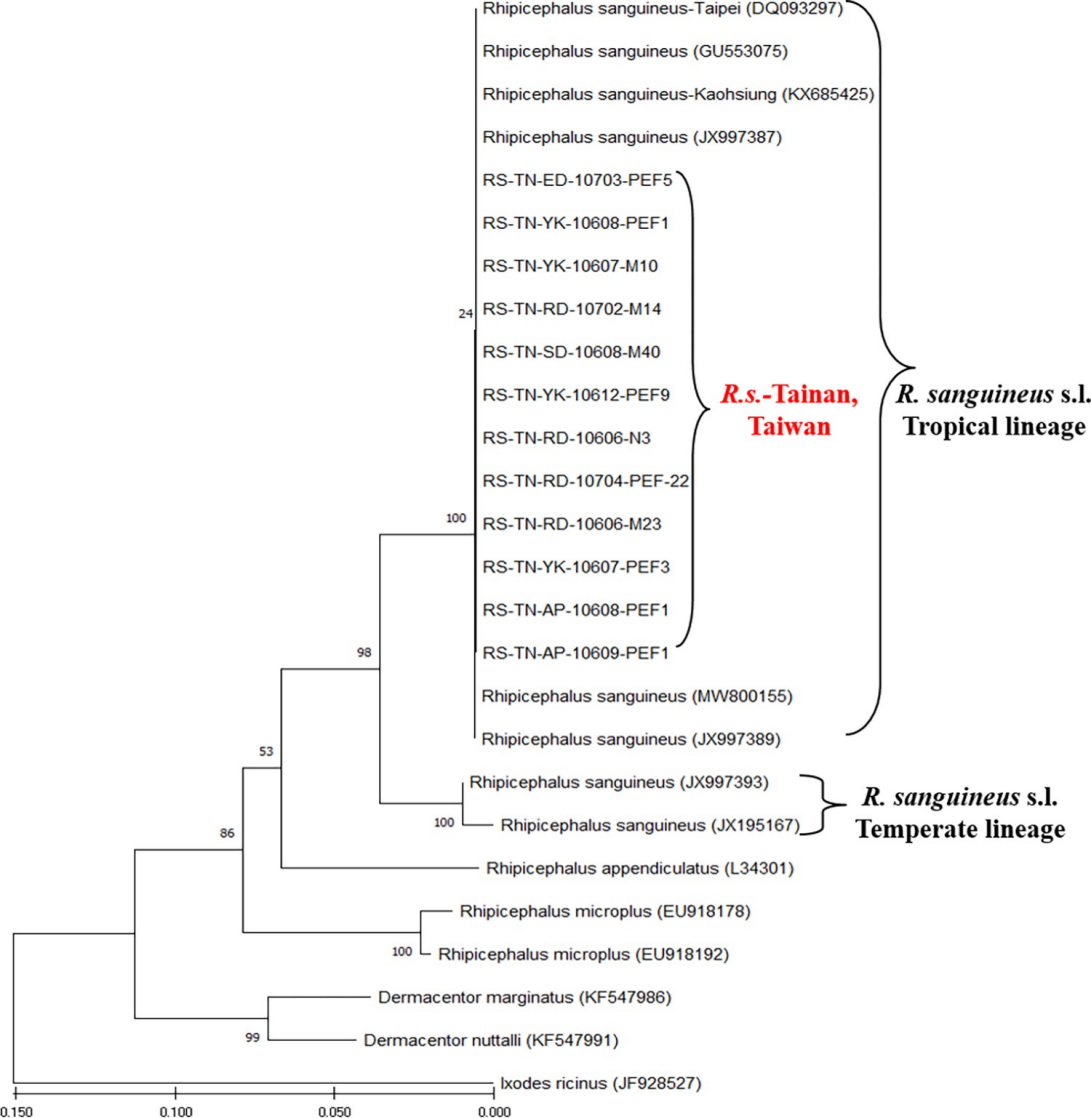
Phylogenetic relationships of *Rh*. *sanguineus* ticks of Tainan based on mitochondrial 16S rRNA gene. Sequences of 12 specimens of *Rh*. *sanguineus* ticks of Tainan were analyzed with 14 other strains of *Rhipicephalus*, *Dermacentor* and *Ixodes* ticks. The tree was constructed and analyzed by neighbour-joining (NJ) method using 1000 bootstraps replicates. Numbers at the nodes indicate the percentages of reliability of each branch of the tree. Branch length is drawn proportional to the estimated sequence divergence.

Molecular analysis of *Rh*. *sanguineus* ticks based on the mitochondrial 16S rRNA gene was performed to demonstrate the genetic relationships among 12 Tainan strains of *Rh*. *sanguineus* and 14 other strains of *Rhipicephalus*, *Dermacentor* and *Ixodes* ticks investigated in this study. Results showed that all the Tainan strains of *Rh*. *sanguineus* ticks are genetically affiliated with the tropical lineage of *Rh*. *sanguineus* sensu lato and can be distinguished from other tick species (**Fig 5**). The submitted sequences of 16S mitochondrial gene were assigned the GenBank accession numbers: ON951615-620 and ON951652-657.

## Discussion

This study provides the first molecular detection and genetic identification of *R*. *massiliae* in *Rh*. *sanguineus* ticks of Taiwan. In previous studies, the *R*. *massiliae* was firstly isolated from a patient in Sicily of Italy [20] and further identified in patients from southern France and Argentina [21, 22]. In addition, *R*. *massiliae* was first isolated from *Rhipicephalus* ticks in Marseilles [47] and is commonly found in *Rh*. *sanguineus* or *Rh*. *turanicus* ticks from France, Greece, Spain, Portugal, Switzerland, Central Africa, and Mali [48, 49]. In this study, *Rickettsia* species detected in *Rh*. *sanguineus* ticks from Taiwan are genetically affiliated to the genospecies of *R*. *massiliae* and *R*. *felis*, and these detections are different from the previous studies which described a *R*. *honei*-like organism in *Ixodes granulatus* ticks identified from Thailand, Taiwan and Japan [31, 32, 37]. Indeed, our study provides the first evidence of *R*. *massiliae* detected in *Rh*. *sanguineus* ticks of Taiwan (Figs 3 and 4) which is considered as an emerging tick-borne pathogen of Spotted Fever Group *Rickettsia*. However, the role of *R*. *massiliae* in public health needs to be further investigated. Thus, our study provides the first molecular evidence and convincing sequences of *R*. *massiliae* in *Rh*. *sanguineus* ticks of Taiwan based on *gltA* (GenBank accession numbers: ON093122-093130) and *ompB* (GenBank accession numbers: ON093131-093138) genes.

The actural mechanism for the transmission of flea-borne *R*. *felis* into *Rh*. *sanguineus* ticks remains elusive. It is possible that the horizontal transmission of *Rickettsia* was occurred between tick-flea interaction. Indeed, ticks may accidentally feed on dogs that were previously fed by infected fleas, and these ticks may acquire the *Rickettsia* infection through feeding blood from the same parasitized dogs [50]. Another possible mode of transmission by co-feeding mechanism may also contributed to the ticks feeding closely with another infected flea on the same host that may facilitate the transmission of pathogens from an infected vector to a new vector [51]. In addition, detection of *Rickettsia* DNA in ticks may not actually demonstrate the viable bacteria in these ticks and also unable to discriminate the source of *Rickettsia* from infested ticks or from blood of dogs. Because of the close contact of dogs with humans, these observations may highlight the epidemiological significance of dogs serving as carrier host for the *Rickettsia* transmission to human population.

Phylogenetic relationships among *Rickettsia* in *Rh*. *sanguineus* ticks can be determined by analyzing the sequence homogeneity of the *gltA* and *ompB* genes of *Rickettsia*. Indeed, sequence analysis based on the *gltA* and *ompB* genes of *Rickettsia* strains among various species from different origins had been shown to be useful for evaluating the genetic relatedness of *Rickettsia* detected from various biological and geographical sources [13, 28–37]. In this study, the phylogenetic analysis based on the sequences of *gltA* and *ompB* genes from *Rh*. *sanguineus* ticks of Taiwan demonstrated a highly genetic homogeneity affiliated to the genospecies of *R*. *massiliae* and *R*. *felis* (Figs 3 and 4). The *R*. *massiliae* strains from Taiwan are mainly associated with the *Rickettsia* strains identified from human patients (GenBank accession no. KJ663741 and KJ663753) and the *R*. *felis* strains are mainly affiliated to the *Rickettsia* strains from cat flea and lice (GenBank accession no. MG893575 and MG818715). In addition, the genetic analysis based on *gltA* also revealed the discrimination of *R*. *massiliae* from the clades composed by *R*. *honei*, *R*. *conorii* and *R*. *rickettsia* as well as by *R*. *japonica*, *R*. *parkeri* and *R*. *sibirica*. The phylogenetic trees constructed by either NJ or ML analysis strongly support the discrimination between the *Rickettsia* strains in *Rh*. *sanguineus* ticks collected from Taiwan and other genospecies of *Rickettsia* from different geographic and biological origins. Thus, the genetic identities of *Rickettsia* strains detected in *Rh*. *sanguineus* ticks of Taiwan were verified as a monophyletic group affiliated to the spotted fever (*R*. *massiliae*) and transitional (*R*. *felis*) groups of *Rickettsia*.

The global climate change may also increase the geographical expansion of ticks that will enhance the transmission of tick-borne pathogens [52]. Indeed, a previous 10-year study on rickettsias conducted in Germany demonstrated that the *Rickettsia* infection rate was significantly increased over the years from 33.3% in 2005 to 50.8% in 2015 [53]. In addition, the previous study also discover that the *I*. *ricinus* tick is reported to have spread into the previously unidentified northern areas of Sweden, Finland and Norway [54]. Because the *Rh*. *sanguineus* ticks are mainly parasitized on dogs that are living closely with residential area of humans. There is a serious concern regarding whether the *Rickettsia* species within this tick species can be transmitted to humans. Thus, further studies focused on the geographical identification of vector ticks and genetic diversity of tick-borne *Rickettsia* may help to illustrate the spread of vector ticks and the risk of transmission of tick-borne rickettsial infections in Taiwan.

## Supporting information

S1 Table Phylogenetic analysis of *Rickettsia* strains used in this study. (DOCX)
