# Supplementary material for: First detection and molecular identification of Rickettsia massiliae, a human pathogen, in Rhipicephalus sanguineus ticks collected from Southern Taiwan
Source: PLoS Negl Trop Dis. 2022 Nov 11;16(11):e0010917. doi: 10.1371/journal.pntd.0010917 (PMC9683588; doi:10.1371/journal.pntd.0010917)
Supplement: S1 Table — (DOCX) [file pntd.0010917.s001.docx]

**S1 Table. Phylogenetic analysis of *Rickettsia* strains used in this study.**

| **Genospecies/strain** | **Origin of *Rickettsia* strain** | | **Gene accession number^a^** | |
| --- | --- | --- | --- | --- |
|  | **Biological** | **Geographic** | ***gltA*** | ***ompB*** |
| Taiwan strains |  |  |  |  |
| RS-TN-ED-10703-PEF5 | *Rhipicephalus sanguineus* | Taiwan | ON093127 | ON093134 |
| RS-TN-YK-10608-PEF1 | *Rhipicephalus sanguineus* | Taiwan | ON093130 | ON093138 |
| RS-TN-YK-10607-M10 | *Rhipicephalus sanguineus* | Taiwan | ON093129 | ON093136 |
| RS-TN-RD-10702-M14 | *Rhipicephalus sanguineus* | Taiwan | ON093128 | ON093135 |
| RS-TN-YK-10607-PEF3 | *Rhipicephalus sanguineus* | Taiwan |  | ON093137 |
| RS-TN-SD-10608-M40 | *Rhipicephalus sanguineus* | Taiwan | ON093125 |  |
| RS-TN-YK-10612-PEF9 | *Rhipicephalus sanguineus* | Taiwan | ON093126 | ON093133 |
| RS-TN-RD-10606-N3 | *Rhipicephalus sanguineus* | Taiwan | ON093123 |  |
| RS-TN-RD-10704-PEF22 | *Rhipicephalus sanguineus* | Taiwan | ON093124 | ON093132 |
| RS-TN-RD-10606-M23 | *Rhipicephalus sanguineus* | Taiwan | ON093122 | ON093131 |
| *Rickettsia massiliae* | Homo sapiens | Italy | KJ663741 |  |
| *Rickettsia massiliae* | Homo sapiens | Italy |  | KJ663753 |
| *Rickettsia massiliae* | Tick | India | MZ851180 |  |
| *Rickettsia massiliae* | Type strain | France |  | AF123714 |
| *Rickettsia massiliae* | Tick | Portugal |  | MW114502 |
| *Rickettsia japonica* | Unknown | France | U59724 |  |
| *Rickettsia japonica* | Unknown | France |  | AF123713 |
| *Rickettsia sibirica* | Unknown | France | U59734 |  |
| *Rickettsia sibirica* | Type strain | France |  | AF123722 |
| *Rickettsia parkeri* | *Amblyomma ovale* | Mexico | MK814825 |  |
| *Rickettsia parkeri* | Type strain | France |  | AF123717 |
| *Rickettsia honei* | Unknown | Australia | AF022817 |  |
| *Rickettsia honei* | Thai tick | France |  | AF123724 |
| *Rickettsia conorii* | Unknown | France | U59730 |  |
| *Rickettsia conorii* | Human blood | Italy |  | JN182798 |
| *Rickettsia rickettsii* | *Dermacentor andersoni* | USA | U59729 |  |
| *Rickettsia rickettsii* | Unknown | USA |  | X16353 |
| *Rickettsia akari* | Human | USA | U59717 |  |
| *Rickettsia akari* | Type strain | France |  | AF123707 |
| *Rickettsia australis* | Human | Australia | U59718 |  |
| *Rickettsia africae* | Tick | Kenya | MH938655 |  |
| *Rickettsia felis* | Cat flea | Malta | MG893575 |  |
| *Rickettsia felis* | Lice | China | MG818715 |  |
| *Rickettsia felis* | Flea | Saint kitts | MT048288 |  |
| *Rickettsia felis* | Tick | Taiwan | MT847616 |  |
| *Rickettsia felis* | Tick | Taiwan | MT847618 |  |
| *Rickettsia felis* | Tick | Chile |  | JF751024 |
| *Rickettsia felis* | Flea | Korea |  | HQ236389 |
| *Rickettsia prowazekii* | Unknown | Unknown | M17149 |  |
| *Rickettsia prowazekii* | Type strain | France |  | AF123718 |
| *Rickettsia typhi* | Type strain | France | U59714 |  |
| *Rickettsia typhi* | Flea | Korea |  | HQ236390 |
| *Rickettsia bellii* | Type strain | France | U59716 |  |
| *Rickettsia bellii* | Unknown | France |  | AY970508 |

**^a^**GenBank accession numbers for *gltA* (ON093122-30) and *ompB* (ON093131-8) were submitted by this study.
